# Knowledge, Attitudes, and Training Needs for AI in Primary Care: National Survey Study of Clinicians in the Veterans Health Administration

**DOI:** 10.2196/90641

**Published:** 2026-04-22

**Authors:** Varsha G Vimalananda, Ben Kragen, Alison J Leibowitz

**Affiliations:** 1 Center for Health Optimization and Implementation Research Edith Nourse Rogers Memorial Veterans Hospital Bedford, MA United States; 2 Chobanian & Avedisian School of Medicine Boston University Boston, MA United States

**Keywords:** artificial intelligence, primary health care, surveys and questionnaires, health personnel, education, professional

## Abstract

**Background:**

Clinicians are the interface between artificial intelligence (AI) applications and patient care. To maximize benefits and minimize risks of AI, clinicians must be “AI-ready”—that is, willing and able to understand, evaluate, and appropriately use AI tools in practice. Prior literature suggests that clinicians lack fundamental competencies in the use of AI. These gaps could be especially problematic in primary care, given its broad reach into patient care. We surveyed primary care providers (PCPs) in the United States’ largest integrated health care system, relatively early in its widespread implementation of clinical AI for frontline use, in order to identify readiness gaps that may warrant particular attention as part of a comprehensive AI implementation strategy.

**Objective:**

The aim of this study was to characterize PCPs’ use, knowledge, attitudes, and training priorities related to AI in order to inform health system AI implementation efforts.

**Methods:**

We conducted a national cross-sectional survey of United States Veterans Health Administration (VA) PCPs in October 2025, assessing AI use, self-reported knowledge, attitudes, and training experience. Descriptive analyses summarized responses with exploratory bivariate comparisons across clinician subgroups. Conventional content analysis with inductive coding was used to characterize open-ended responses providing a definition of AI.

**Results:**

Among 170 respondents (170/989, 17.2% response rate), 66.5% (113/170) reported current AI use, most commonly generative AI and decision support tools. Overall attitudes toward AI were positive, with 70.6% (120/170) mostly enthusiastic or more enthusiastic than apprehensive. Confidence in understanding sources of AI bias (62/170, 36.5%) and ethical issues (81/170, 47.6%) was limited. When asked to define AI, very few respondents provided an accurate technical definition. Key concerns about use of AI included accountability, accuracy, and transparency. Though 88.2% (150/170) identified AI training as a priority, only 26.5% (45/170) had any training. Training experiences ranged widely in source, focus, and structure.

**Conclusions:**

PCPs are eager to harness AI’s practical advantages but lack foundational competencies to do so in ways that maximize benefit and minimize risk. Our findings highlight a need for targeted education that prioritizes critical appraisal, workflow integration, and risk mitigation, supported by governance that addresses clinicians’ concerns and validated measures to evaluate progress toward an AI-ready workforce. These steps can empower PCPs to leverage AI safely and effectively and strengthen the quality and safety of primary care delivery at scale.

## Introduction

Clinician readiness for artificial intelligence (AI) in health care is critical to realizing AI’s potential in practice. Clinicians are the interface between AI applications and patient care. They are responsible for interpreting and integrating AI outputs within the complex realities of individual patients, incomplete information, and clinical uncertainty. Even when AI systems are “trustworthy,” with rigorous risk assessment and mitigation built in, vulnerabilities remain at the point of clinical application where judgment, workflow integration, and accountability ultimately reside. To maximize benefits and minimize risks, clinicians must be “AI-ready”—that is, willing and able to understand, evaluate, and appropriately use AI tools in practice, as reflected by their knowledge and attitudes related to clinical AI.

AI introduces a unique set of concerns for clinicians, including accountability for errors, algorithmic bias, and potential erosion of the patient-clinician relationship [1,2]. Without sufficient knowledge or supportive attitudes, clinicians may fail to realize AI’s benefits. But of greater concern are threats to clinical quality, safety, ethics, patient trust, and clinician well-being. Recognizing these risks, health systems and professional organizations have identified an AI-ready clinical workforce as a top strategic priority [3,4].

AI use in clinical care is expanding rapidly, including ambient documentation [5], image analysis, and point-of-care clinical guidance. However, existing literature suggests clinicians have limited understanding of AI fundamentals, data and algorithmic bias, and ethical and legal implications, alongside concerns about workflow integration and clinician-patient relationships [6-9]. These gaps may be particularly problematic in primary care. Primary care providers (PCPs) are the first point of contact for most patients and interact with a wide range of clinical, operational, and administrative technologies. Limited readiness among PCPs could therefore have broad implications for patient safety and quality of care.

We conducted a national survey of PCPs in the Veterans Health Administration (VA) to describe current AI use, characterize knowledge and attitudes regarding AI in clinical care, and identify priority areas for education and implementation support. We also explored variations in knowledge and attitudes across PCP subgroups. Findings on readiness components are intended to inform health system efforts to ready PCPs for safe, effective AI use.

## Methods

### Setting and Sample

The VA is the largest integrated health system in the United States, with over 9.1 million enrollees across 1380 facilities [10]. We conducted a cross-sectional survey of VA PCPs in October 2025. All VA PCPs were eligible. Using the VA Corporate Data Warehouse, we randomly selected 1000 PCPs and emailed invitations to complete an online survey using MS forms, with 2 reminders; 989 PCPs were successfully contacted. Participants were informed that identifying information would be used solely to link responses to demographic data and would be blinded for analysis.

### Survey Development

Survey development was guided by Rogers’ Diffusion of Innovation Theory, which positions user knowledge and attitudes as key determinants of technology adoption [11]. We aimed to understand PCP readiness rather than the implementation context as a whole (ie, the organizational context for use). To develop the survey, we first reviewed measures in the literature that have been used to measure knowledge and attitudes toward AI among various groups, including the general population, employees, medical students, and health professionals [12-20]. None were well-suited to our goal of using a brief survey to measure high-level knowledge about clinical AI or attitudes toward use of AI in clinical care specifically. For example, measures were too detailed in their assessments of AI literacy for a group that has not undergone a formal curriculum [12,13], focused on negative attitudes toward AI [14-17] or examined user experience [18-20] with a single tool. We therefore drafted knowledge items based on topics commonly measured in literacy surveys that might reasonably be assessed via self-report, common attitudes about use of AI widely reported in the literature, and potential outcomes from use of AI in primary care. The initial pool of 20 items was reviewed, refined, and reduced to 14 items in total with input from AI and clinical leadership at the national and regional level (n=4). The final survey included an item assessing current AI tool use, 2 closed-ended and one open-ended item on self-assessed AI knowledge, 4 items on attitudes toward AI, and 4 items on training experience and priorities. Knowledge questions were intended as indicators of foundational AI literacy, and attitude questions captured perceived benefits and risks relevant to AI in clinical care.

### Data Analysis

We calculated descriptive statistics and compared survey respondents to nonrespondents on age, gender, rurality, and clinical credential (physician vs other credential), using VA Corporate Data Warehouse data. We summarized patterns in AI use, knowledge, attitudes, and training priorities. Open-ended responses to the question asking for a definition of AI were analyzed using conventional content analysis. An inductive coding approach was used, allowing categories to emerge directly from the data rather than a predetermined framework. This process involved repeated reading of responses to categories of responses with iterative refinement into broader categories to characterize the range and depth of respondents’ understanding of AI concepts.

No inferential tests were performed given the exploratory nature of the study; however, we conducted planned bivariate comparisons across clinician subgroups to inform system-level targeting of training and implementation support. We examined whether responses on the 2 knowledge questions (“I feel confident describing potential sources of bias in AI applications” and “I have a good grasp of the ethical issues that may arise in my use of AI;” Strongly Agree/Agree vs Strongly Disagree/Disagree/Neither Agree nor Disagree) and on the overall attitude question (“What is your overall attitude toward using AI tools in your work as a PCP?”; Mostly Enthusiastic/More Enthusiastic than Apprehensive vs Mostly Apprehensive/More Apprehensive than Enthusiastic/Equally Enthusiastic and Apprehensive) differed by age (continuous variable), rural practice setting (yes/no), current use of any AI tools (yes/no), and prior AI training (yes/no).

### Ethical Considerations

This project was determined to be quality improvement and not human subjects research by the VA Bedford Healthcare System Institutional Review Board.

## Results

### Participants’ Characteristics

A total of 170 PCPs completed the survey (170/989, 17.2% response rate). Respondents and nonrespondents did not differ significantly on demographic characteristics. The majority of the respondents were aged 40 to 59 years (110/170, 64.7%), female (115/170, 67.6%), and physicians (105/170, 61.8%); about one-third practiced in rural locations (Table 1). One-third (52/170, 30.6%) considered themselves among the earliest adopters of new technology in general, with another 51.2% (87/170) considering themselves among early (but not the earliest) adopters. Nearly half (80/170, 47.1%) of the respondents used VA GPT (the VA’s internal generative AI chat tool), 37.1% (63/170) used OpenEvidence (an AI-enabled clinical decision support platform), 11.2% (19/170) used Microsoft Copilot, and 33.5% (57/170) reported no current AI use.

**Table 1:** Demographic characteristics of the respondents (N=170).

Characteristic, category		Values, n (%)
**Age group (y)**		
	30-39	27 (15.9)
	40-49	52 (30.6)
	50-59	58 (34.1)
	60-69	29 (17.1)
	70-79	3 (1.8)
	Missing	1 (0.6)
**Rural practice location**		
	Rural	53 (31.2)
	Nonrural	117 (68.8)
**Gender**		
	Female	115 (67.6)
	Male	55 (32.4)
**Clinical credential**		
	Physician (MD/DO)	105 (61.8)
	Non-physician clinician (Nurse Practitioner, Physician Assistant, etc)	65 (38.2)
**Current use of artificial intelligence tools^a^**		
	No current use	57 (33.5)
	VAGPT	80 (47.1)
	OpenEvidence	63 (37.1)
	Ambient scribing/dictation using VAGPT	14 (8.2)
	Microsoft Copilot	19 (11.2)
	Other artificial intelligence tools	12 (7.1)
**General stance toward new technology**		
	First to try, willing to take risks	52 (30.6)
	Embrace early after seeing potential impact	87 (51.2)
	Wait until technology has proven value	24 (14.1)
	Skeptical and cautious, adopt after most others	6 (3.5)
	Last to adopt, prefer traditional approaches	1 (0.6)
**Formal or informal artificial intelligence training**		
	No training	125 (73.5)
	Yes training	45 (26.5)

^a^“Check all that apply” item; respondents could select multiple artificial intelligence tools.

### Knowledge of AI

Confidence describing potential sources of bias in AI was mixed: 36.5% (62/170) agreed or strongly agreed that they were confident, 41.8% (71/170) were neutral, and 21.2% (36/170) disagreed. Confidence in grasping ethical issues arising from AI use was somewhat higher, with 47.6% (81/170) agreeing or strongly agreeing, 32.9% (56/170) neutral, and 18.8% (32/170) disagreeing or strongly disagreeing. Increasing age was positively associated with more confidence describing potential sources of bias.

Respondents were asked “Based on your current level of familiarity, how would you define AI technology in a few words?” The breadth of content across responses ranged widely, containing elements of definitions but also descriptions of how AI works, use cases, perceived impact, and attitudes toward AI. While very few responses were technical definitions that referenced AI’s ability to simulate human intelligence, one participant defined AI as “A computer system designed to perform tasks that typically require human intelligence such as reasoning, learning, and decision making. It works through algorithms and analyzing databases.” When definitions were provided, they were more often nontechnical: “a system that uses data and engineering to produce solutions.” Several responses defined AI via a use case: “A way to summarize the visit and create a template for review to save time on tedious documentation.” Others defined AI in terms of impact, both positive “a time saver” and negative “horrific as it is a HUGE concern for patient privacy.” Still others simply expressed a lack of knowledge: “Not sure of exactly what it is. My guess is like ChatGPT that it can help with writing, looking for clinical diagnosis, etc.”

### Attitudes Toward AI

Most respondents expressed positive attitudes toward AI tools in clinical work: 38.8% (66/170) were mostly enthusiastic, 31.8% (54/170) more enthusiastic than apprehensive, and 18.8% (32/170) equally enthusiastic and apprehensive. Only 3.5% (6/170) were mostly apprehensive. A more positive overall attitude toward AI was associated with current use of AI, but not age, rurality, or AI training.

**Figure 1:**
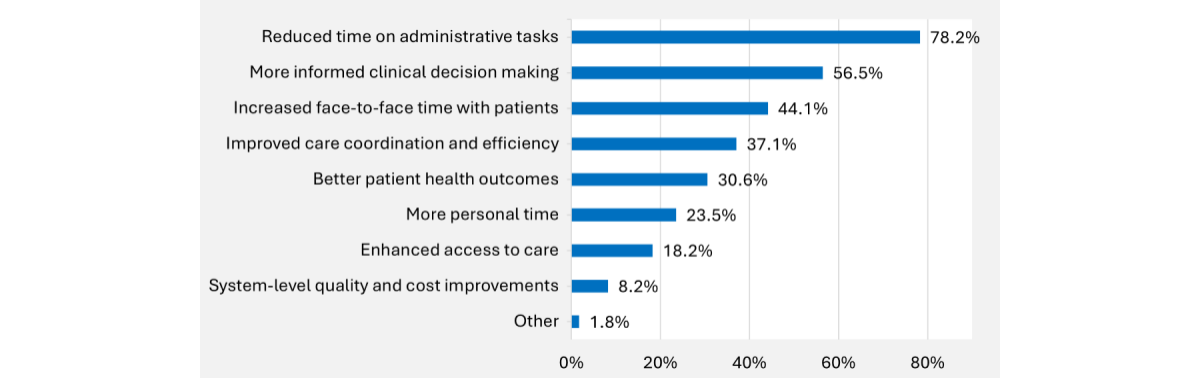
Positive outcomes of artificial intelligence in clinical care about which primary care providers are most enthusiastic (top 3 choices). Bars represent the percentage of respondents who selected each option among their top 3 choices; percentages do not sum to 100%.

When asked to select the 3 positive outcomes about which they were most enthusiastic, respondents most frequently selected reduced time on administrative tasks (133/170, 78.2%), more informed clinical decision-making (96/170, 56.5%), and increased face-to-face time with patients (75/170, 44.1%) (Figure 1).

**Figure 2:**
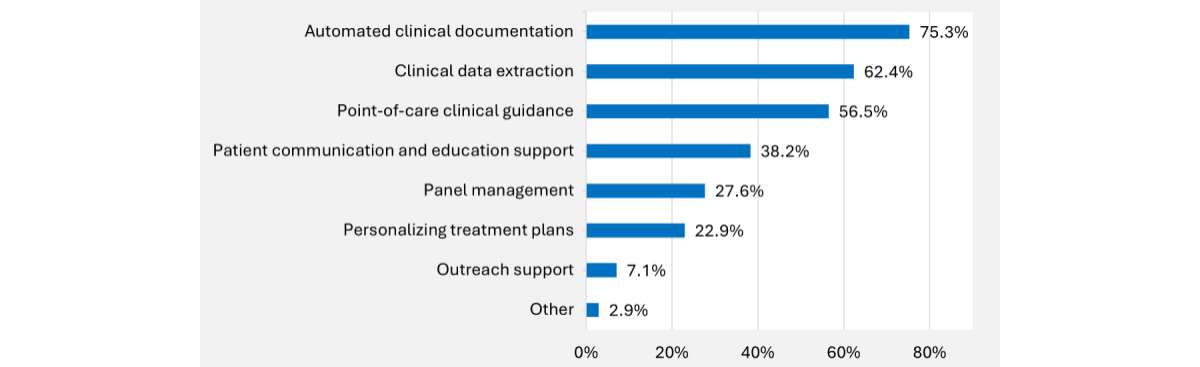
Use cases for artificial intelligence in clinical care about which primary care providers are most enthusiastic (top 3 choices). Bars represent the percentage of respondents who selected each option among their top 3 choices; percentages do not sum to 100%.

Respondents were also asked to indicate the three use cases about which they were most enthusiastic (Figure 2). Most frequently selected were automated clinical documentation (128/170, 75.3%), clinical data extraction (106/170, 62.4%), and point-of-care clinical guidance (65/170, 38.2%).

**Figure 3:**
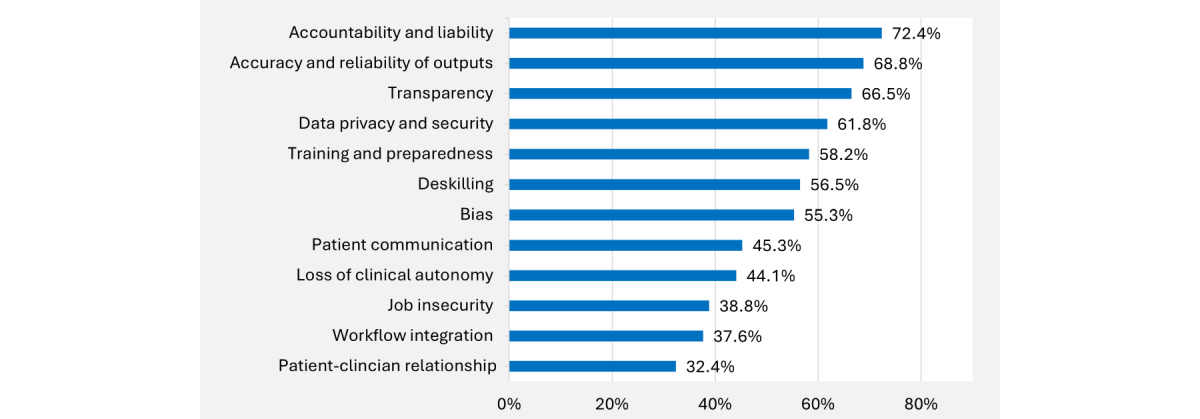
Potential issues raised by artificial intelligence in clinical care about which primary care providers are most apprehensive. Bars represent the percentage of respondents who selected “moderately apprehensive” or “very apprehensive” for the listed issues.

Regarding potential concerns, respondents were “moderately apprehensive” or “very apprehensive” about accountability and liability (123/170, 72.4%), accuracy of outputs (117/170, 68.8%), and transparency in how AI reached a decision (113/170, 66.5%) (Figure 3). Of less concern were job security (66/170, 38.8%), workflow integration (64/170, 37.6%), and impact on patient-clinician relationships (55/170, 32.4%).

### Training Experience and Priorities

Only 26.5% (45/170) reported having any formal or informal AI training, with sources ranging from YouTube and in-service sessions to Google AI and formal coursework. Training was a moderate or high priority for 88.2% (150/170). For their top 3 training priorities, 78.2% (133/170) selected workflow integration; 58.8% (100/170) selected how to evaluate output for accuracy, safety, and bias; and 49.4% (84/170) selected tool-specific training. Foundational AI knowledge (74/170, 43.5%) and ethical, legal, and regulatory issues (78/170, 45.9%) were also commonly prioritized. Communication about AI with patients was least frequently prioritized (17/170, 10%).

Taken together, these results show that PCPs are actively using and optimistic about AI tools, yet experience notable gaps in foundational knowledge, evaluative skills, and training exposure.

## Discussion

This national survey of VA PCPs reveals a promising foundation for successful AI implementation in primary care. Respondents expressed strong enthusiasm for tools that could improve workflow efficiency and enhance decision-making, especially in high-value use cases such as automated documentation, data extraction, and point-of-care guidance. Many respondents had already adopted AI chat or decision support tools. These findings highlight meaningful openness to AI innovation in primary care and suggest that health systems could capitalize on existing clinician interest and experimentation with AI at the front lines.

At the same time, respondents reported uneven knowledge about AI fundamentals and limited confidence addressing bias and ethical issues, which are domains central to safe and responsible AI use. Qualitative responses underscore the limited technical understanding of AI among many clinicians. Apprehension regarding liability, accuracy, and transparency suggests that clinicians recognize key risks and feel ill-equipped to evaluate or mitigate them. Training experience was limited and heterogeneous despite nearly universal interest. Together, these findings illuminate a tension, which is that PCPs are eager to harness AI’s practical advantages but lack foundational competencies to do so in ways that maximize benefit and minimize risk.

Our results align with prior studies showing clinicians’ rapid AI uptake alongside uncertainty around the implications of use, including technical evaluation, risk mitigation, and professional responsibilities [6-9]. Our study extends existing research by focusing on primary care—a setting with broad clinical reach and substantial implications for quality and safety—and by examining a single, integrated health system actively pursuing AI readiness. By identifying specific gaps in knowledge, attitudes, and training, our findings point to levers that health system leaders directly influence through implementation support and governance.

The VA and other health systems have prioritized developing an “AI-ready” workforce [21]. Our findings suggest concrete steps toward this goal. Standardized, clinician-focused training on foundational AI concepts, paired with policies requiring baseline AI literacy and tool-specific training, could help establish consistent expectations and competency across a health system. Many states mandate continuing medical education in high-impact areas such as end-of-life care, risk management, and electronic health record usage. Given that AI carries comparable implications for patient safety, quality of care, and workflow, similar mandatory educational requirements may be warranted as AI’s role in care delivery expands. Validated measures of AI readiness could further support needs assessment, targeted training and implementation efforts, and evaluation over time. Beyond education, governance structures can establish expectations for transparency, accountability, and workflow integration.

Concerns that were lower ranked on the survey remain important for comprehensive AI implementation, such as communicating about AI with patients, considering impact on patient-clinician relationships, and using AI for outreach support and population health management. Respondents may have prioritized these concerns lower because they are less immediate than daily workflow demands. Nonetheless, focused educational content can raise awareness of these important issues without overwhelming core training needs. A tiered approach to training could address immediate workflow and safety concerns first while introducing relational and population health considerations as AI use matures in practice.

Although this study focuses on VA primary care, several findings likely generalize beyond this setting. VA PCPs practice within an integrated health system that offers relatively consistent access to new technologies, training resources, and technical support. These features may facilitate earlier exposure to AI tools compared with some non-VA settings where access to these resources may be more variable. At the same time, our findings suggest that even in a system with substantial organizational capacity, clinicians still report uneven foundational knowledge and limited preparedness to evaluate potential risks, suggesting that these challenges may be at least as notable in more resource-constrained environments. Similar findings have been reported outside of the United States, showing that clinicians across diverse health systems express enthusiasm for AI but face gaps in readiness [17,22,23]. The parallels within the VA context and outside of it emphasize a broader need for health systems to invest in developing AI readiness among their clinicians in the interest of supporting safe and effective AI use.

This study has limitations. While our response is consistent with other VA clinician surveys [24-26] and respondents resembled nonrespondents demographically, technologically enthusiastic respondents may be overrepresented. Knowledge gaps and apprehensions in the population of nonrespondents may be even more pronounced, which would only emphasize the importance of readiness efforts to optimize clinicians’ AI use. Also, our subgroup analyses are intended to be hypothesis-generating rather than definitive; future studies should examine subgroup differences with finer grain.

VA PCPs demonstrate strong enthusiasm for AI tools promising workflow efficiencies and clinical support and report current use of generative AI and decision support technologies. However, they also report limited training and uneven knowledge in foundational and evaluative domains. These findings highlight a need for targeted education that prioritizes critical appraisal, workflow integration, and risk mitigation, supported by governance that addresses clinicians’ concerns and validated measures of progress toward an AI-ready workforce. By providing one of the first system-wide assessments of PCP’s AI readiness within an integrated health system that is itself relatively early in its AI implementation journey, this study offers novel insights into how frontline clinicians are engaging with AI, where critical gaps in readiness exist, and which levers health systems can use to enable safe and effective AI adoption that optimizes patient care while minimizing risks. Addressing these gaps will enable health systems to capitalize on clinician openness while strengthening the quality and safety of primary care delivery at scale.

## Data Availability

The datasets generated or analyzed during this study are available from the corresponding author on reasonable request.

## Abbreviations

AI: artificial intelligence
PCP: primary care provider
VA: Veterans Health Administration
